# Histology and chronological magnetic resonance images of congenital spinal deformity: An experimental study in mice model

**DOI:** 10.1186/s12891-024-07460-8

**Published:** 2024-04-26

**Authors:** Haruki Ueda, Takuya Iimura, Satoshi Inami, Hiroshi Moridaira, Takuya Yazawa, Yoshiteru Seo, Hiroshi Taneichi

**Affiliations:** 1 Department of Orthopaedic Surgery, Dokkyo Medical University School of Medicine, 880 Kitakobayashi, Mibu, Shimotsuga, Tochigi, Japan; 2 Department of Pathology, Dokkyo Medical University School of Medicine, 880 Kitakobayashi, Mibu, Shimotsuga, Tochigi, Japan; 3 Department of Homeostatic Regulation, National Institute for Physiological Sciences, 38, Nishigonaka, Myodaiji, Okazaki, Aichi Japan

**Keywords:** Congenital spinal malformation, Chronological follow-up, Histological and MR imaging analysis, Spinal malformation mouse

## Abstract

**Background:**

The natural history of the congenital spinal deformity and its clinical magnitude vary widely in human species. However, we previously reported that the spinal deformities of congenital scoliosis mice did not progress throughout our observational period according to soft X-ray and MRI data. In this study, congenital vertebral and intervertebral malformations in mice were assessed via magnetic resonance (MR) and histological images.

**Methods:**

Congenital spinal anomalies were chronologically assessed via soft X-ray and 7 T MR imaging. MR images were compared to the histological images to validate the findings around the malformations.

**Results:**

Soft X-ray images showed the gross alignment of the spine and the contour of the malformed vertebrae, with the growth plate and cortical bone visible as higher density lines, but could not be used to distinguish the existence of intervertebral structures. In contrast, MR images could be used to distinguish each structure, including the cortical bone, growth plate, cartilaginous end plate, and nucleus pulposus, by combining the signal changes on T1-weighted imaging (T1WI) and T2-weighted imaging (T2WI). The intervertebral structure adjacent to the malformed vertebrae also exhibited various abnormalities, such as growth plate and cartilaginous end plate irregularities, nucleus pulposus defects, and bone marrow formation. In the chronological observation, the thickness and shape of the malformed structures on T1WI did not change.

**Conclusions:**

Spinal malformations in mice were chronologically observed via 7 T MRI and histology. MR images could be used to distinguish the histological structures of normal and malformed mouse spines. Malformed vertebrae were accompanied by adjacent intervertebral structures that corresponded to the fully segmented structures observed in human congenital scoliosis, but the intervertebral conditions varied. This study suggested the importance of MRI and histological examinations of human congenital scoliosis patients with patterns other than nonsegmenting patterns, which may be used to predict the prognosis of patients with spinal deformities associated with malformed vertebrae.

## Background

Congenital spinal deformity is a spinal abnormality caused by vertebrae that are not properly formed or segmented. This pathology is not infrequent, with a reported incidence of 3–50 in 100,000 births [1–4]. The natural history of this deformity has been reported, and the progression of the deformity and its clinical magnitude vary depending on the type, number, and location of the malformed vertebrae [5–8]. Animal models of this anomaly have been generated, and several substances and environmental factors are known to induce this condition, including hypoxia, carbon monoxide, insulin, and alcohol [9–12]. The involvement of several genes has been reported, and they are recognized as multifactorial and include both environmental and genetic factors [13, 14]..

We previously reported the creation and chronological observation of a congenital scoliosis mouse model via magnetic resonance (MR) microscopy [15]. In that report, we presented three congenital spinal abnormalities in two mice. According to soft X-ray and MRI data, the spinal deformities of these mice did not progress throughout our observational period. Although MRI showed an abnormal vertebral shape and a narrowed intervertebral space adjacent to the malformed vertebra, MRI alone did not provide us with detailed local information or clues as to why the deformities did not progress. For example, structural alterations in growth plate have to be assessed as one of the possible causes [16, 17], which could not be clarified by our MRI. In this report, congenital spinal anomalies were histologically assessed, and MR images were compared with histological data for validation.

## Methods

Housebred DBA-1 J mice were utilized as the congenital scoliosis model. The detailed methodology used to create the mouse model and to obtain the soft-X-ray and MR images is described elsewhere [15]. Newborn mice with spinal malformations were subjected to follow-up with repetitive soft X-ray and MR images, and the malformed vertebrae were histologically evaluated. The Digital Imaging and Communications in Medicine (DICOM) data of the MR images were viewed by 3D multiplanar reconstruction in Horos (ver3.3.6, www. horosproject.org), creating the coronal slice corresponding to the histology sample. This project was approved by the Animal Research Councils of our institution (judgment reference number 988). This study was carried out in compliance with the ARRIVE guidelines. Soft X-rays were taken under sedation with Isoflurane, and MRI were taken under general anesthesia using sevoflurane with monitoring the vital signs. After the final MRI session, the mice were euthanized by increasing the sevoflurane until they stopped breathing for a sufficient time.

### MRI protocol

MR images were obtained by a 7 Tesla microimaging system (AVANCE III, Bruker BioSpin, Ettlingen, Germany) with a 1 Hz radiofrequency (RF) surface coil (16 mm in diameter, for 7Tesla, 300 MHz, Doty Scientific, Inc., SC, USA). The typical parameters used for three-dimensional T1-weighted gradient-echo imaging (3D-T1WI) via fast low angle shot (FLASH) were as follows: 19.2 × 19.2 × 9.6 mm field of view (FOV), 192 × 192 × 96 data matrix, 50 ms repetition time (TR), 3.75 ms echo time (TE), and a flip angle of 22.5°. Three-dimensional T2-weighted rapid acquisition with relaxation enhancement (RARE) imaging (3D-T2WI) was conducted with the same voxel resolution with the parameters of TR/TE/RARE factor = 1000 ms/50 ms/16. The final voxel resolution was 50 × 50 × 50 μm.

### Histological preparation

At sacrifice, the entire spine was removed, fixed in 10% neutral buffered formalin (pH 7.4) for 1 week and decalcified in 10% EDTA (pH 7.4). The malformed parts of the spine with adjacent discs and vertebrae were embedded in paraffin and cut into coronal sections 5 μm in thickness by a microtome. The sections were double stained with Alcian blue followed by hematoxylin and eosin. The sections were analyzed under a light microscope (Olympus BX53).

## Results

Two mice born to mothers exposed to carbon monoxide (CO) were found to have spinal malformations. Two additional mice with spinal malformations were born to non-CO-exposed mothers. MR images of the middle thoracic region or above were blurry due to respiratory motion; hence, we observed four malformations in the distal thoracic spine of four mice.

### Normal control

**Fig. 1:**
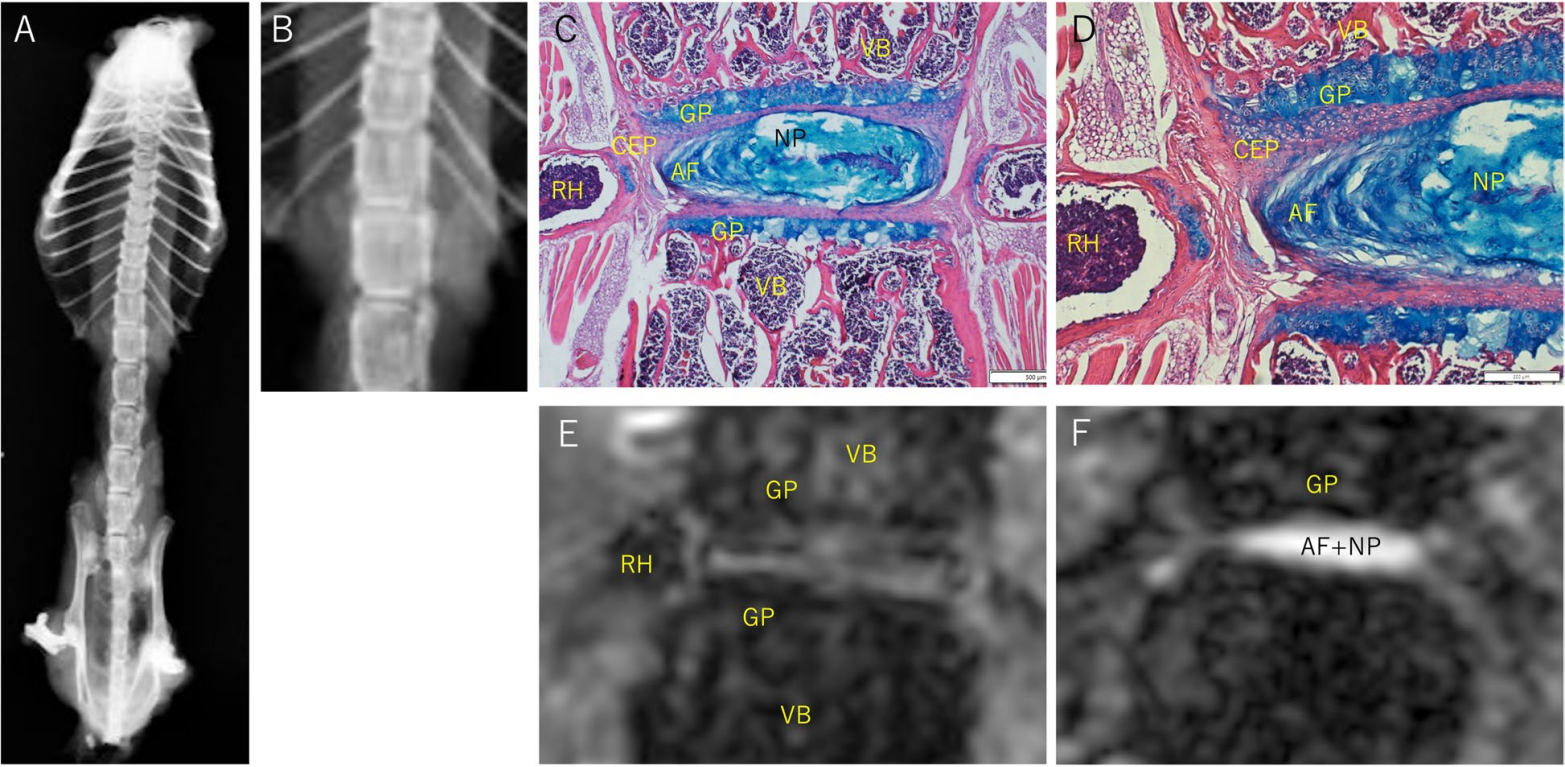
Normal control (female mouse, 67 weeks old at the MRI, specimen Xray, and histology). A, B: Soft X-ray images of thoracic and lumbar spine and pelvis **A** and the thoracolumbar spine **B**. The cortical surface of the vertebrae is shown as high density on the side of the vertebrae, and the cancerous bone of the vertebrae is less dense than the cortical surface. The intervertebral disc space is shown as a darker line. The growth plate at the edge of the bony vertebrae appears as a higher density line because of calcareous deposition. **C**, **D**: Histology around the intervertebral space of thoracolumbar spine (C × 40, D × 100). The cartilaginous end plate (CEP) is stained in light red and contains some blue chondrocytes, and there is no bony end plate. Between the CEP and vertebral body (VB), the growth plate (GP) is stained blue. The GP is approximately 100 μm thick and is seen as a blue layer consisting of structural cell layers of proliferating, prehypertrophic, and hypertrophic chondrocytes with insular light blue zones. Between the two CEP layers the nucleus pulposus (NP) and the annulus fibrosus (AF) are observed in the thick lentoid light blue compartment, spreading nearly the full width of the vertebrae. **E**: T1-weighted MR image corresponding to histology. Osseous structures, especially cortical bone and trabecular bone, have very low intensity. GP is observed to have a linear low intensity. Among the GPs, NP and AF and CEP had slightly greater intensities. **F**: T2-weighted MR image. Thc contour of the structures are less clear. The combination of NP and AF has a very high intensity in the intervertebral space and is considered a water-rich structure. CEP, cartilaginous end plate; VB, Vertebral body, AF, annulus fibrosus; NP, nucleus pulposus; RH, rib head

The soft X-ray, histological and MR images of a coronal slice of a normal spine are shown as a normal control (67-week-old female mouse) in Fig. 1.

A soft X-ray image of the thoracic and lumbar spine and the pelvis exenterated from a normal adult mouse is shown. A pair of symmetrical ribs were attached to the proximal end of each thoracic vertebra. The craniocaudal length of the vertebra increased from the proximal thoracic spine to the distal lumbar spine. The whole alignment of the spine and the contour of each vertebra are depicted, and intervertebral spaces between vertebrae are shown as dark lines.

Histologically, the vertebrae and intervertebral space are shown. The growth plate (GP), cartilaginous end plate (CEP), nucleus pulposus (NP), and annulus fibrosus (AF) were stained, and their borders were very clear. The bony end plate does not exist in mice [18].

On T1WI, the osseous structures of the vertebrae and ribs were observed to exhibit dappled low intensity, and the cortical bone surface had very low intensity. The dappled appearance of the vertebral body likely corresponds to a mixture of the trabecular bone, which is a hard calcified tissue and exhibits very low intensity on MRI, and the bone marrow, which contains more fat and water and appears to have relatively higher intensity. The GP, in which the cartilage matrix is calcified and endochondral ossification occurs, appears as a low-intensity line demarcating the vertebra from the intervertebral space. The intervertebral spaces were defined as slightly higher-intensity flat band transecting vertebrae. Based on the shape, this high-intensity band seemed to include the NP, AF, and CEP. The high-intensity line between the rib head (RH) and vertebrae is either a cartilaginous or a fibrous structure. On T2WI, osseous structures were observed at low intensity. A very high-intensity lentoid shape lay between the vertebrae. This high signal intensity and lentoid shape are consistent with the water-rich structure of the combination of the NP and AF.

### Mouse 1 distal thoracic spine

**Fig. 2:**
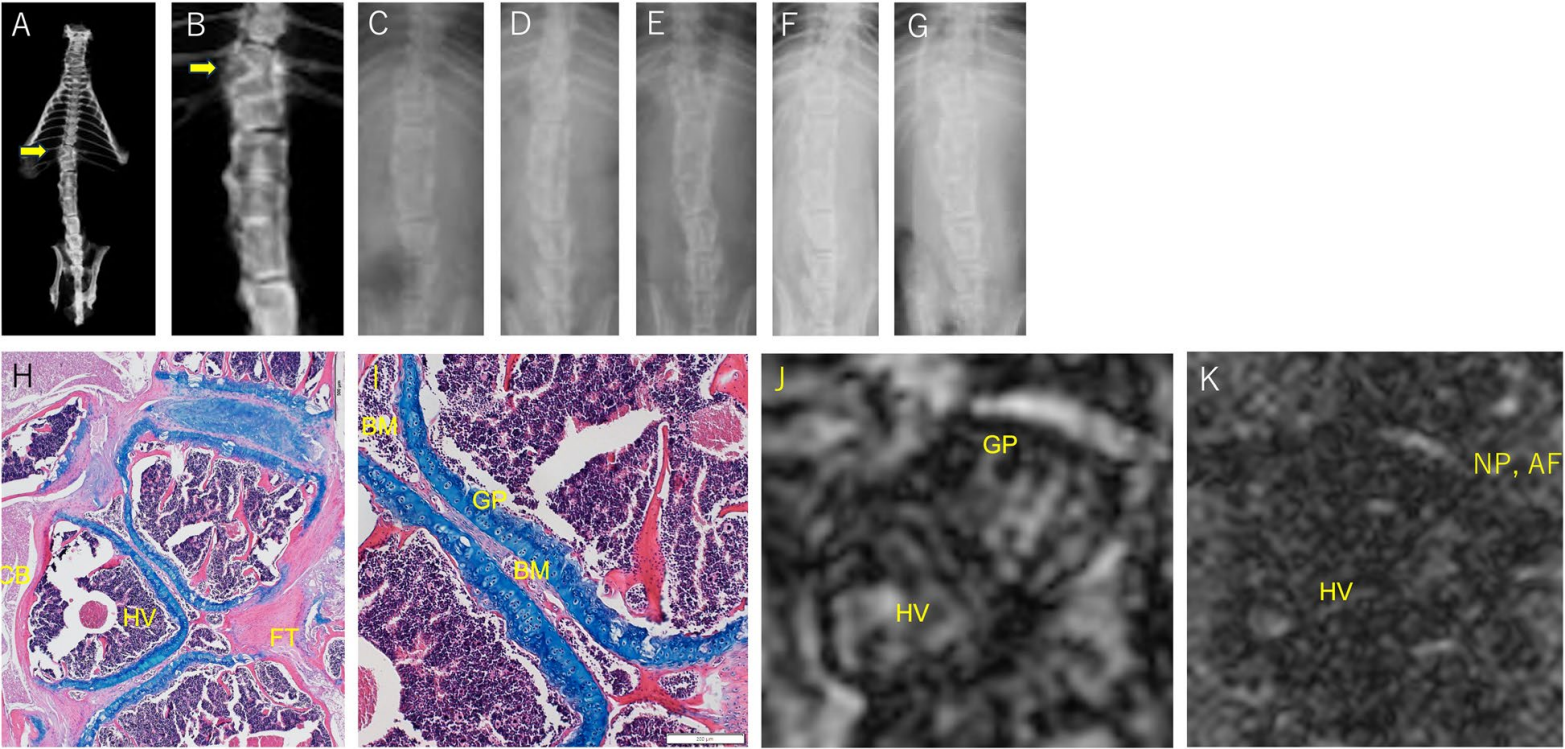
Mouse 1, distal thoracic spine (male mouse, 30.5 weeks old at MRI, specimen X-ray, and histology). A-G: Soft X-ray images. **A**, **B**: specimen, **C**-**G**: 5.5 weeks, 11.5 weeks, 14 weeks, 17 weeks, and 23 weeks after birth, respectively. There is a block of abnormal vertebrae with a triangular hemivertebra (HV) shown by the arrow. The border lines between vertebrae are seen as high-density lines, but the intervertebral disc space cannot be seen. **H**, **I**: Histology (H × 40, I × 100). The HV has one cortical bone surface stained in red and two GP surfaces stained in blue. The wedging vertebrae adjacent to the HV are framed by the GP. The intervertebral space surrounding the HV is very thin, containing the CEP and bone marrow (BM). The GP around the HV is thinner with random hypertrophic chondrocytes, and the layers of the cells are more irregular than those in the normal tissue. On the concave side, thick fibrous tissue (FT) of the costovertebral joint capsule is formed, bridging the adjacent malformed vertebrae. **J**: T1-weighted MR image. The dark low-intensity line surrounding the HV and the wedging vertebra corresponds to the GP or cortical bone, and the slightly high intensity line between the HV and the wedging vertebra corresponds to the insufficiently formed CEP and BM. The dark line of the GP borders the osseous vertebrae and FT of the costovertebral joint capsule, which has a high intensity. On T2WI, the contour of the osseous structures is unclear, but the normal NP and AF cranial to the wedging vertebra appear at higher intensity. No high-intensity structure were detected between the HV and wedging vertebra, which is consistent with the lack of NP and AF observed in the histological images. **K**: T2-weighted MR image. The normal NP and AF cranial to the wedging vertebra appear at higher intensity, while no bright signal is observed around the HV. Hemivertebra (HV), bone marrow (BM), cortical bone (CB), fibrous tissue (FT)

A block of malformed vertebrae at the distal thoracic spine was observed in a CO-exposed mouse (Fig. 2).

The distal thoracic spine of this mouse had a block of malformations, including a hemivertebra (HV) and wedging vertebra. Histologically, the intervertebral space surrounding the HV was very thin and contained bone marrow (BM), suggesting osseous fusion around the hemivertebra. Compared with those around the normal tissue, the GPs around the HVs were thinner, and the layers of the cells were irregular and fewer. The intervertebral space above the wedging vertebra, which contained the NP and AF surrounded by the CEP, appeared normal.

T1WI and T2WI of MR images corresponding to the histology sections are shown. The T1WI showed that the contour of the structure was sharper. On T1WI, the wedging vertebra and hemivertebra were observed to contain rich bone marrow, which is more intense on MRI. The fibrous tissue on the concave side also exhibited greater intenisty. On T2WI, the shape of the osseous structures was unclear. No high-intensity structures were observed between the HV and wedging vertebra, which is consistent with the lack of histological images of the NP and AF in these regions.

### Mouse 2 distal thoracic spine

**Fig. 3:**
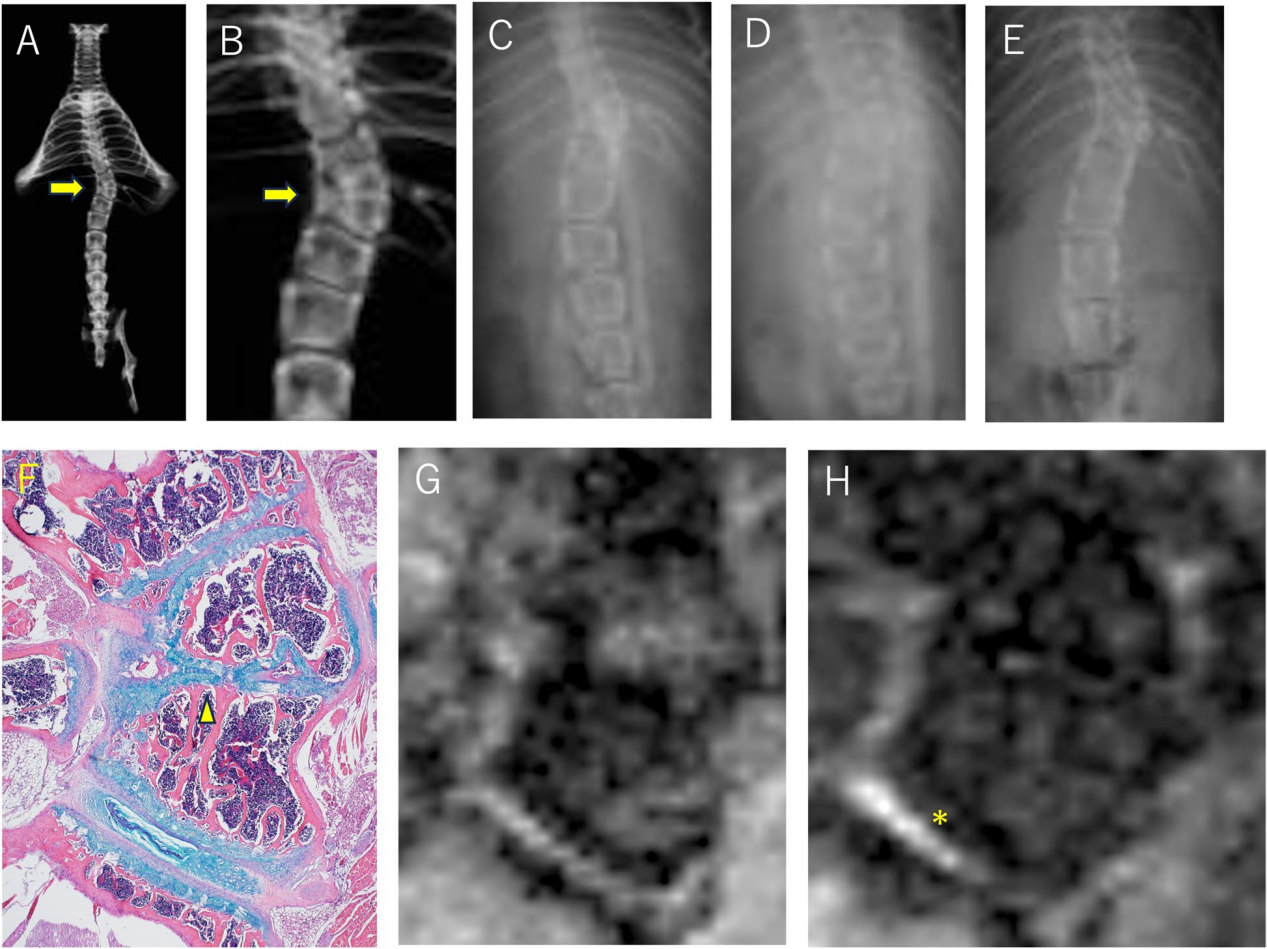
Mouse 2, distal thoracic spine (male mouse, 11.5 weeks old at MRI, specimen X-ray, and histology) A-E: Soft X-ray images. **A**, **B**: specimen, **C**-**E**: 3.5 weeks, 7.5 weeks, 12 weeks after birth respectively. Wedging vertebrae at the distal thoracic spine are demarcated by high-density lines, but the intervertebral structures involved are unclear. **F**: Histology, × 40. Malformed vertebrae have more trabecular bone on the concave side and more bone marrow on the convex side. GPs of the malformed vertebrae are meandering and irregular in thickness, containing mostly hypertrophic chondrocytes, and partially merged with adjacent GPs (arrowhead). The CEP and BM are observed in the space between these irregular GPs, but the NP is lacking. On both the concave and convex sides of the curve, the thick FT of the costovertebral joint capsule covers the surface of the malformed segment. **G**: T1-weighted MR image. Malformed vertebrae are surrounded by a low-intensity band, which corresponds with the irregular width of the GP observed via histology. The convex side with rich bone marrow was observed at a higher intensity. **H**: T2-weighted MR image. The caudal intervertebral space is brightly intense, which is consistent with the presence of the NP and AF according to histology(*)

Mouse 2 was another CO-exposed mouse with malformed vertebrae in the thoracolumbar region (Fig. 3).

On the X-ray images, two adjoining wedge-shaped vertebrae were observed in the distal thoracic spine of mouse 2. These two adjacent small wedge vertebrae caused local scoliosis. Histologically, the malformed vertebrae had irregular GPs, and the intervertebral space around them was abnormal. The CEP and BM were observed in the space between these irregular GPs, but the NP was lacking. The caudal intervertebral space of the caudal malformed vertebra contained the CEP, AF and NP.

The malformed vertebrae are contoured by black bands on T1W MR images, which is consistent with the observations in GPs. No high-intensity area in the intervertebral space between the malformations was observed on either the T1WI or T2WI. Thick fibrous tissue (FT) over the concave surface exhibited high intensity on T1WI and was distinguishable from the cortical bone surface. Histologically, a caudal intervertebral space with a CEP, AF and NP was observed to have a high intensity on T1WI from side to side and a bright high intensity on T2WI, which is consistent with the appearance of a normal intervertebral structure.

### Mouse 3 Hemivertebrae in the proximal lumbar spine

**Fig. 4:**
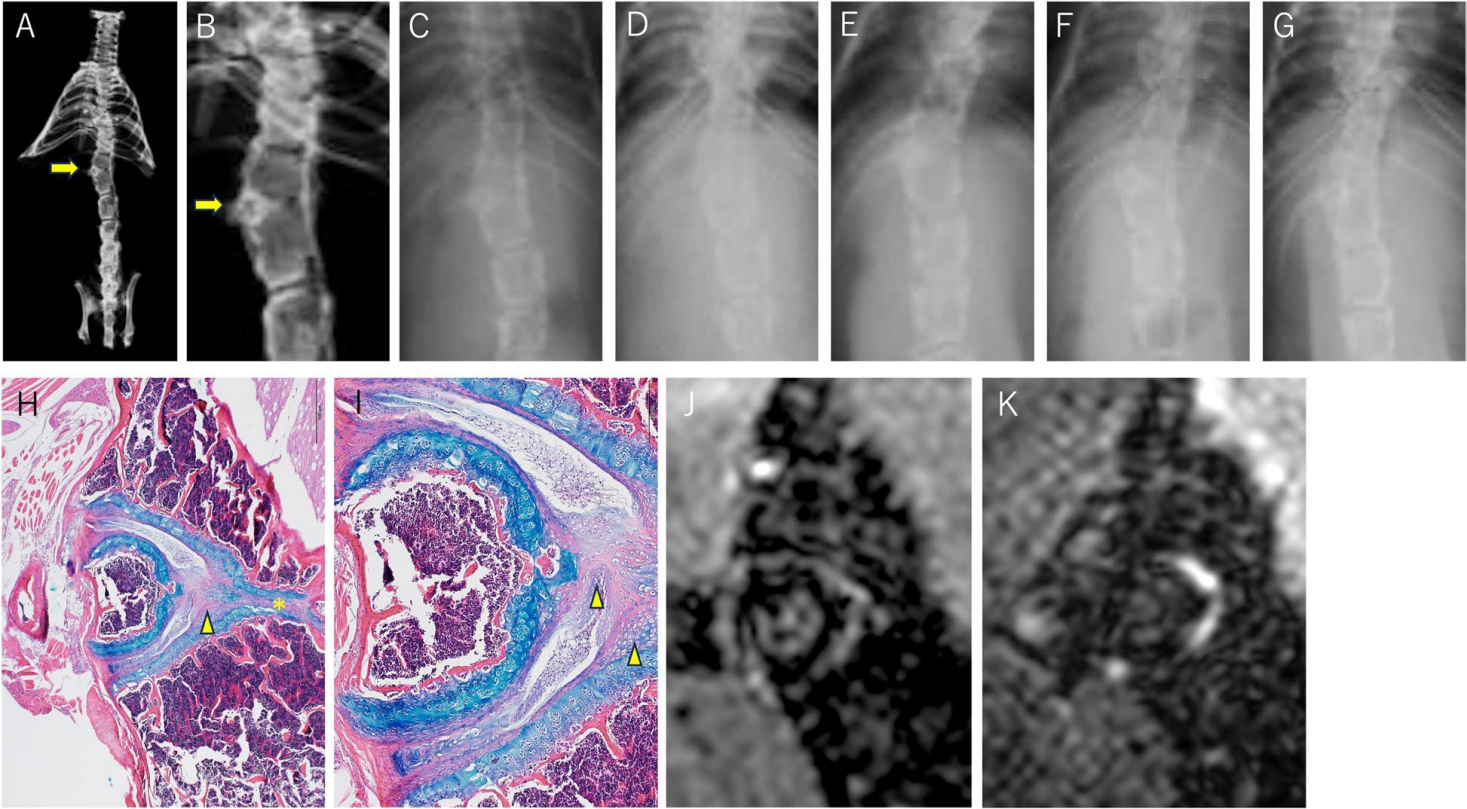
Mouse 3, Hemivertebra in the proximal lumbar spine (female mouse, 31.5 weeks old at the MRI, specimen X-ray, and histology) A-G: Soft X-ray images. **A**, **B**: specimen, **C**: 10 weeks, **D**: 13 weeks, **E**: 16 weeks, **F**: 22 weeks, and **G**: 27 weeks after birth. The HV is shown (arrow). In the middle thoracic spine, there are multiple malformed vertebrae with conjoined ribs. **H**, **I**: Histology of the hemivertebra (H × 40, I × 100). Approximately two-thirds of the HV surface is covered with the structured GP, and the remaining one-thirds is covered with cortical bone. The intervertebral space surrounding the HV contains normal intervertebral structures, including the NP, AF, and CEP. In the two NPs, young immature cells are observed, and the development of the NP seems to be delayed. The two NPs are separated by the CEP on the right side of the HV (arrowhead). Two vertebrae confining the HV on the convex side adjoin each other on the concave side. The GPs look thinner and insufficiently formed, and there is a CEP on these GPs but no NP or AF is observed (*). **J**: T1-weighted MR image. The contour of the HV is shown as a dark low signal intensity corresponding to the GPs and cortical bone. The hemivertebral body is slightly more intense, showing a bone marrow-rich content. The intervertebral space surrounding the HV was observed at high intensity. **K**: T2-weighted MR image. The bright high-intensity area is limited to the right part of the intervertebral space around the HV, which corresponds to the NP in the histology

On soft X-ray, Mouse 3 had multiple malformed vertebrae with conjoined ribs in the thoracic spine and an HV in the proximal lumbar spine (Fig. 4).

The HV in the thoracolumbar spine had a short rib, causing local scoliosis. Spinal deformities did not develop during the observation period. Histologically, the HVs had structured GPs that were approximately 100 μm in thickness and covered approximately two-thirds of their surface. The intervertebral space surrounding the HV contained normal intervertebral structures, including the NP, AF, and CEP. Two vertebrae adjacent to the HV faced each other on the concave side, between which there was a CEP and a thinner GP, but no NP or AF was observed.

In the MR images, the GP and intervertebral structures were distinguishable according to the corresponding signal intensity. Specifically, the presence of the NP and AF was clearly indicated by the bright high intensity on T2WI.

### Mouse 4 wedging vertebrae in the distal thoracic spine

**Fig. 5:**
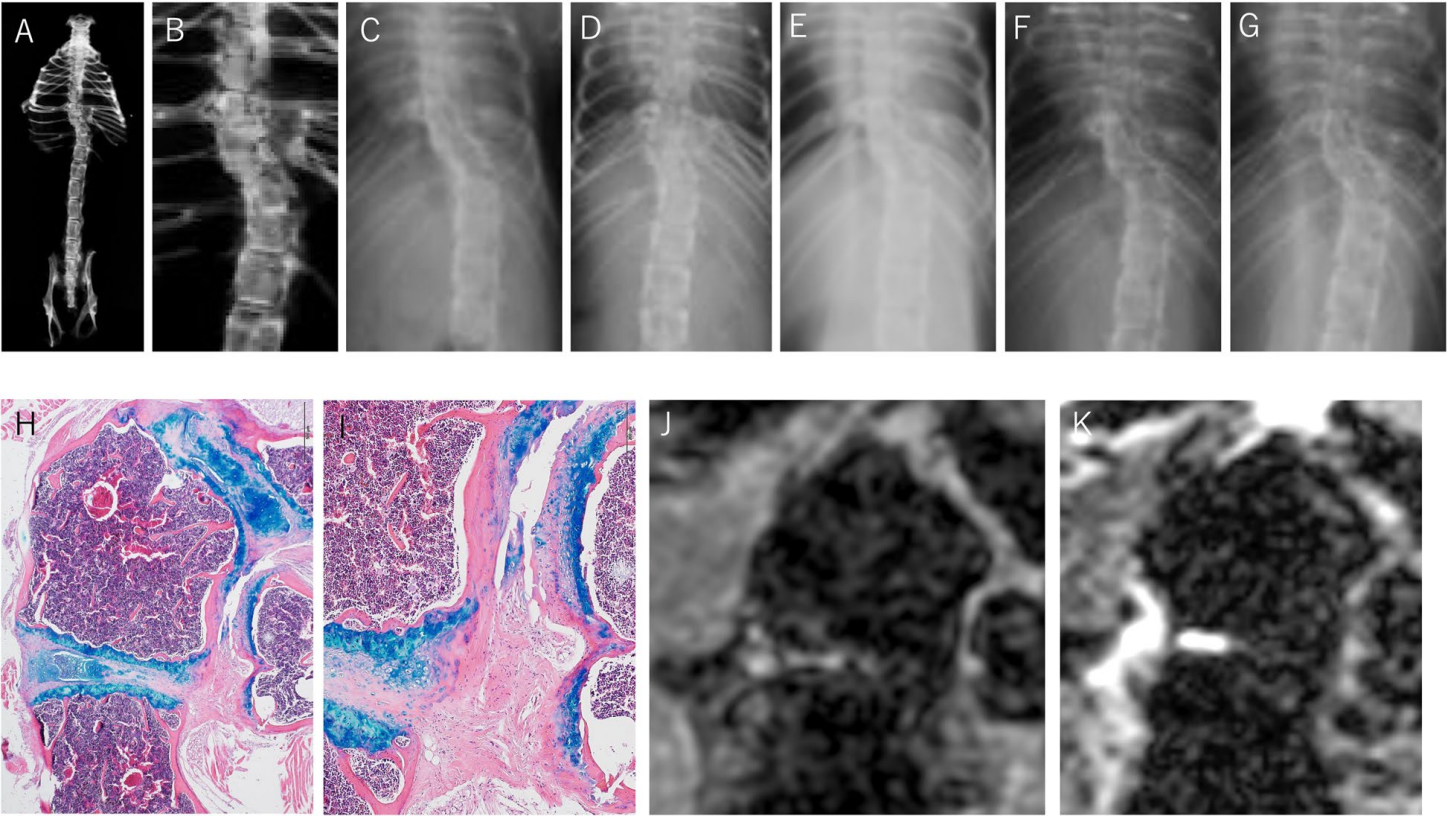
Mouse 4, Wedging vertebra with a conjoined rib (male mouse, 78 weeks old at the MRI and 80 weeks old at specimen Xray and histology) **A**-**G**: Soft X-ray images. **A**, **B**: specimen, **C**: 10 weeks, **D**: 13 weeks, **E**: 16 weeks, **F**: 22 weeks, and **G**: 27 weeks after birth. Steep sigmoid deformity was observed but did not develop during the observation period. Intervertebral spaces are difficult to see in the X-ray images of the living mouse. **H**, **I**: Histology of the wedging vertebra (H × 40, I: joint between vertebra and conjoined rib, × 100). In the adjacent intervertebral space, the NP, AF, and CEP were observed. Conjoined ribs have a large RH and no cortex or GP. A thick fibrous structure occupies the space between the vertebra and the RH. **J**, **K**; T1-weighted and T2-weighted MR images. In both images, the abnormal RH is well demarcated. High-intensity cranial and caudal intervertebral disc space is observed on T1WI, and the bright high-intensity area on T2WI consistently indicates the presence of the NP. Histologically, the region between the rib heads and vertebrae is filled with fibrous connective tissue. This region is observed at high intensity in both T1WI and T2WI

Mouse 4 had a sigmoid deformity in the thoracolumbar spine (Fig. 5).

On soft X-ray film, several sets of unsegmented multiple ribs were observed on both sides in the thoracolumbar region. Histologically, there was a wedging vertebra with unsegmented abnormal ribs on its concave side. The adjacent intervertebral space contained normal structures, although they were not in good shape. A fibrous connection was observed between the concave side of the spine and the abnormal ribs.

In the T1WI, the osseous structures of the vertebral body and the abnormal rib heads were observed at low intensity and were clearly demarcated. High-intensity regions between the rib heads and vertebrae were observed in both the T1WI and T2WI. This signal pattern is consistent with the fibrous connection at the costovertebral joint in the normal control group.

### Chronological comparison via MRI

**Fig. 6:**
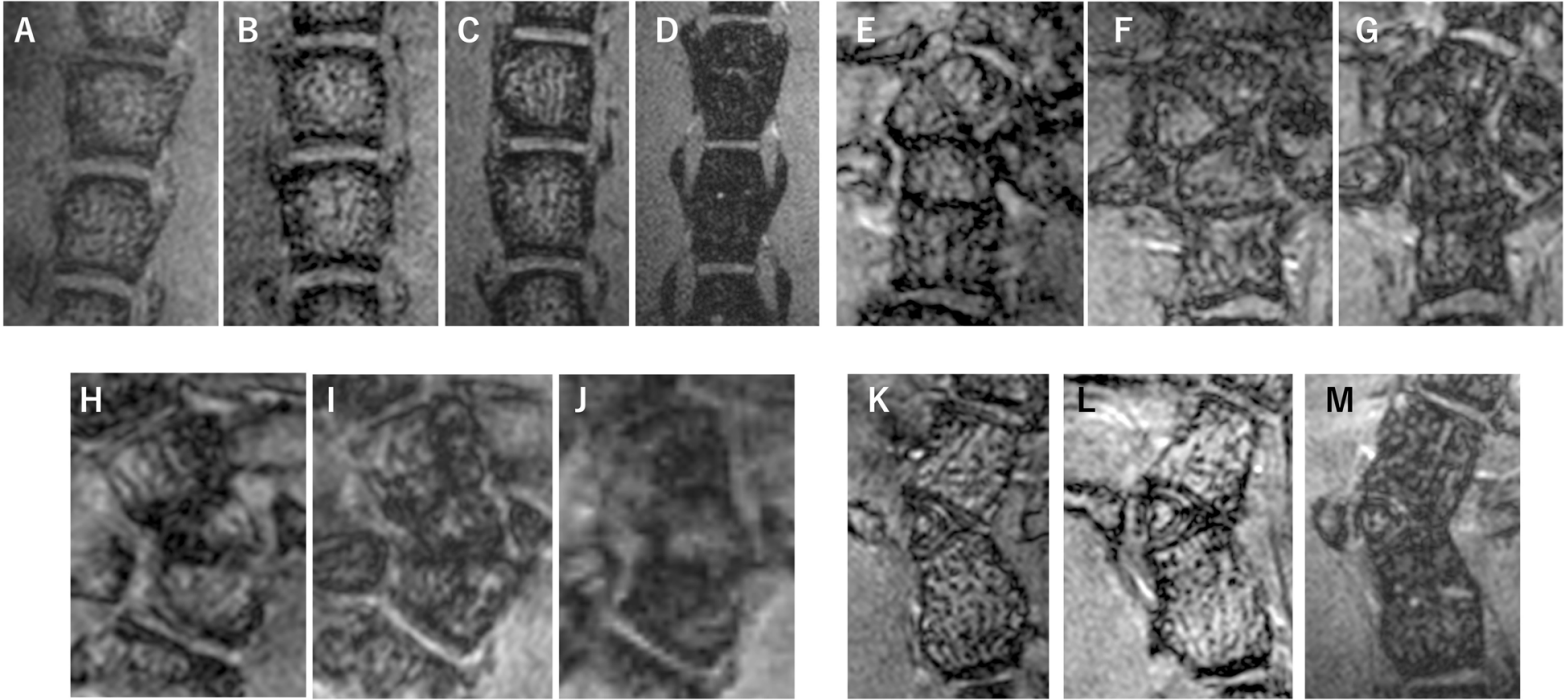
Chronological comparison of T1-weighted MR images **A**-**D**: Normal mouse at 4.4 weeks, 5.5 weeks, 7 weeks, and 37 weeks after birth, respectively. **E**-**G**: Mouse 1 at 8 weeks, 26 weeks, and 30 weeks after birth, respectively. **H**-**J**: Mouse 2 at 6 weeks, 9 weeks, and 12 weeks after birth, respectively. **K**-**M**: Mouse 3 at 14 weeks, 26 weeks, and 32 weeks after birth, respectively. In the normal mouse, the vertebrae seem vertically longer in the postgrowth MRI (D) compared to those in the maturing period (A-C). However, the shape and thickness of the structures did not change over time. The stripe in the vertebrae appears thicker over time and may indicate the development of the bony trabecular structure

In Fig. 6, serial T1WI of a normal mouse during and after maturation and of mouse 1, mouse 2, and mouse 3 are chronologically shown.

The shape of the vertebrae in the normal mouse was vertically longer on postgrowth MRI but otherwise appeared to be similar. The signal intensity of the intervertebral spaces and the thickness of the cortical bones or GPs did not change. The stripe appearance in the vertebrae appeared to thicken over time, which may indicate the development of the bony trabecular structure.

## Discussion

We previously observed congenital spinal malformations in mice via soft X-ray films and 7 T MRI [15]. In that study, we showed the ability of this method to finely depict normal and abnormal morphology of the mouse spine but could not define the anatomical structures, especially in the malformed region. In this report, the malformed vertebrae were histologically analyzed to validate the MR images and to understand the changes that occurred in the congenital lesion through careful comparisons with histology.

Soft X-ray images displayed the existence of abnormal bony structures by revealing the cortical bone and GP as contours but could not distinguish the presence of the NP or CEP in the intervertebral space. Moreover, 7 T MRI, using the combination of T1WI and T2WI, could distinguish small structures in the mouse spine, for example, GPs approximately 100 μm in thickness or those containing NPs in the intervertebral space. Histological comparison revealed that T1WI distinguished the GP from the osseous vertebra as a low-intensity band and depicted the intervertebral cartilaginous structures as a high-intensity area, and T2WI was sensitive for detecting the NP.

### Deformity progression

In human congenital scoliosis, the progression of the deformity depends on the type, number, and location of the malformed vertebrae [6, 8]. Additionally, the presence of intervertebral structures with growth potential is one of the key factors in deformity progression. Hemivertebrae were classified into three types: fully segmented (a disc on both sides), semisegmented (a disc on one side but the other side being fused to the adjacent vertebra), and nonsegmented (fused on both sides to the adjacent vertebrae). [6–8]All of the malformed vertebrae in this study were fully segmented on soft X-ray, and the spinal deformity could have progressed but did not. One reason is the deficit in the growth potential of the GP adjacent to our malformed vertebrae. In the human study, the alteration of the chondrocytes and their zonal structure in GP were considered possibly affecting the curve development [16, 17]. According to our observations, the structures of the intervertebral spaces of the segmented abnormal vertebrae were not normal, although they were recognized as spaces on soft X-ray images. The abnormalities were diverse and included the thickness and alignment of the GP, the thickness of the CEP, the presence or absence of the NP, and bone marrow formation. The GPs in the malformed vertebrae are thinner and not tidily structured and may lack the normal ability to grow. In addition, some of the abnormal vertebrae could be fused with adjacent vertebrae despite their full segmentation on X-ray images. BM was observed in the narrow intervertebral space adjacent to the hemivertebra of Mouse 1 and the wedge vertebrae of Mouse 2. In a histological study on human congenital scoliosis, Shapiro stated that without normal disc material, cartilage endplates from adjacent vertebrae can merge and establish bony unions between two separate centers well before skeletal maturation [19]. According to his description, the malformed vertebrae in our study lacked adjacent normal disc material, shareed CEP cells with adjacent vertebrae between their irregular GPs, and may have spontaneously fused in the animal’s early life even if they were fully segmented when the animals were born. Another reason could be the difference in the postural mechanism between bipedal and quadrupedal gait on scoliosis development reported by Machida [20]. Our model mice all had quadrupedal defects, and their deformities were considered less likely to progress according to his conclusion, although his scoliosis model was not congenital but rather idiopathic.

### Embryological background

In our spinal malformation model, although the cell distribution was irregular and the structures were dysplastic, no atypical cells were observed. These findings indicate that the malformations observed in the present study likely resulted from abnormalities in cell migration and/or apoptosis rather than from abnormalities in cell differentiation. Two of the four vertebral malformations lacked adjacent NPs. The two abnormalities of the lack of NP and the abnormality in cellular migration can be explained embryologically by notochordal dysfunction since the notochord serves as both the resource of the NP and the critical signaling center for the formation of the axial skeleton [21–23]. Notochord cells move into intervertebral regions, proliferate and undergo hypertrophy to form the NP [24–26]. Prior to this process, the signal from the notochord induces somite differentiation to the sclerotome, which subsequently gives rise to the AF, GP, and vertebral bodies [23]. Defective and small condensations of the sclerotome may result in abnormally small or malformed bone [27, 28]. Notably, as an example of a mouse with functional defects in the notochord, Danforth’s short-tailed mouse is known to exhibit early reduction and fragmentation of the notochord, which induces cellular degeneration in the sclerotome and leads to reduced vertebral bodies and short tails [26]. In addition, the dysfunctional effect of the maternal CO exposure on the embryonic notochord is thought as evanescent. The axial spine develops in cranio-caudal sequence and the critical period for the somite development is at day 8.5 for cervical, 9 for thoracic, and 10 for lumbosacral vertebrae [24]. In our study as well as the previous study, the most frequent anomaly formation was seen in thoracolumbar spine and by CO exposure on 9–9.5th day of gestation [11, 15, 29]. This seems to suggest that the CO exposure promptly affected the function of the notochord in the critical period for the thoracolumbar spinal formation, and stopped disturbing the function soon after the finish of exposure without causing multiple vertebral malformations in the caudal spinal column.

In this report, congenital spinal deformities in mice were histologically assessed, and chronological MR images were validated. To our knowledge, chronological and precise observations of individual mice with congenital scoliosis have not been reported in the literature. Moreover, we recognize that these deformities did not progress, and the reason for this lack of progression was discussed. The interbody abnormalities associated with vertebral malformation in humans have not been investigated in detail. The results of this study suggested the importance of MRI and histological examination of human congenital scoliosis lesions other than nonsegmented patterns, which may be used to determine the prognosis of patients with spinal deformities associated with malformed vertebrae.

A limitation of this study is the small number of specimens. The incidence of spinal anomalies in mice is much lower than that in the literature [11, 12, 29]. In prior research, perinatal fetuses or neonates were harvested and sacrificed, but we started screening mice more than 3 weeks after birth to avoid cannibalization by mothers. However, some babies, especially those with anomalies, could have been cannibalized [30]. Additionally, this sporadic harvest of model mice cannot easily increase the number of specimens needed due to ethical considerations. We evaluated vertebral malformations but did not scrutinize minor anomalies, including the posterior column of the spine, because of the visual difficulty associated with identifying small, thin structures in MR and histology images; therefore, small structural anomalies may have been missed. Another limitation of this report is the lack of molecular genetic analysis, which could have provided evidence of the inability of the deformed vertebrae to grow on growth plates. The random imaging timing was another limitation. We could not determine when or how bony fusion around the malformation had occurred. This change could be degeneration due to abnormal mechanical stress or a protective reaction to prevent symptomatic deterioration, and chronological observation using other modalities, including CT scans, would be helpful; however, answering this question is beyond the scope of this study.

## Conclusions

Congenital spinal malformations of mice were histologically analyzed, and MR images were assessed. By combining T1WI and T2WI, 7 T MRI could be used to distinguish small structures around vertebrae. The spinal deformities of the mice did not progress, possibly due to the lack of growth potential of the abnormal growth plate or the ossification of the abnormal intervertebral space adjacent to the malformed vertebrae.

## Data Availability

The datasets used and/or analyzed during the current study are available from the corresponding author upon reasonable request.

## Abbreviations

MR: Magnetic resonance
MRI: Magnetic resonance imaging
WI: Weighted image
GP: Growth plate
CEP: Cartilaginous end plate
NP: Nucleus pulposus
AF: Annulus fibrosus
RH: Rib head
CO: Carbon monoxide
HV: Hemivertebra
BM: Bone marrow
FT: Fibrous tissue
